# Going Abroad

**Published:** 1889-12-21

**Authors:** 


### Going Abroad.

An idea prevails that most invalids would secure a better climate by going abroad for the winter than by remaining on the British Isles, and this may be admitted. When change of climate is recommended from England, the principal object is to remove the patient from raw, damp, foggy weather. There is, however, probably no more favourable climate in the world for the Anglo-Saxon, whether ill or well, than that of England in the summer of an ordinary year. And when a better climate is sought in the winter, the one that approaches nearest to the English summer will, in the majority of instances, be the best. Much caution, however, is necessary in fixing on the place to go to. A person who journeys to many continental health resorts ought to take health with him instead of searching for it when there. The fatigue of the journey, the predilection or otherwise for continental life, the bustle of a crowded hotel, the absence of homely seclusion, the difficulty of obtaining a sick dietary, the separation from friends, the frequent want of good house or neighbouring sanitation, the changes of temperature to which even the most favoured spots are liable, and the expense, are all matters to be taken into consideration. Warmth, dry atmosphere, a blue sky, sunshine and flowers (as on the Riviera in winter) are very good things in their way. But there are drawbacks even to the Riviera. There is a hot sun and a cold shade, there is a great evening fall of temperature, there are periodical cold northerly winds from the distant icy mountains, and there are often musquitos. If, in addition, the invalid is under bad sanitary conditions, he may, perhaps, not live to regret the change. Doubtless many would be better in the end by remaining surrounded by the comforts of home, than by flying to ills yet undiscovered. Neither must the return be forgotten. Invalids having once gone into a warmer climate, are almost compelled to remain there until the "leafy month of June" gives assurance of warmth here. And thus many perforce remain longer abroad than they contemplated when leaving home. We are of opinion that very sick persons should not desert the comforts of home for either hotels or lodgings. Also that, as a general rule, persons disliking continental life should not go abroad. Thirdly, we would not advise persons going who cannot afford to spend money enough, not only to procure everything necessary, but also everything desirable. Each case demands attentive consideration, not only as regards the ailment and a suitable climate for the ailment, but also as respects habits, ideas, idiosyncrasy, temperament, constitution, and means.

